# Multiple and synergistic deregulations of apoptosis-controlling genes in pancreatic carcinoma cells

**DOI:** 10.1038/sj.bjc.6601330

**Published:** 2003-10-28

**Authors:** A Trauzold, S Schmiedel, C Röder, C Tams, M Christgen, S Oestern, A Arlt, S Westphal, M Kapischke, H Ungefroren, H Kalthoff

**Affiliations:** 1 Molecular Oncology, Clinic for General Surgery; 2 Laboratory of Molecular Gastroenterology, 1st Dept. of Medicine, UK S-H, Campus Kiel, Germany

**Keywords:** apoptosis resistance, pancreatic adenocarcinoma, death receptors

## Abstract

Inability to die by apoptosis is one of the reasons for the deregulated growth of tumour cells and the frequently observed failure of chemotherapy. In this study we thought to identify the common and functionally important characteristics responsible for the apoptosis resistance of pancreatic tumour cells. We analysed cell surface expression level of death receptors CD95 and TRAIL-R1-4 as well as the expression profile of sixteen apoptosis-relevant proteins in five pancreatic carcinoma cell lines Capan1, Colo357, PancTuI, Panc89 and Panc1. These data were evaluated in the context of sensitivity towards anti-CD95 and TRAIL-mediated apoptosis. Here we report that except for resistant Panc1 cells, which only marginally expressed CD95, all other cell lines showed comparable levels of CD95 and TRAIL receptors irrespectively of their apoptotic phenotype. Interestingly, we found that the elevated expression of FLIP, Bcl-x_L_ and IAP in parallel with a downregulation of FADD and Bid was common for the resistant cell lines. Consequently, stable overexpression of XIAP, Bcl-x_L_ or dominant negative FADD in sensitive cells significantly reduced the death receptor mediated apoptosis while the overexpression of Bid rendered the resistant cells sensitive.

CD95, TRAIL-R1 (tumour necrosis factor-related apoptosis inducing ligand-receptor 1) and TRAIL-R2 are members of the TNF-receptor family of transmembrane proteins that are capable of inducing apoptosis (Wiley *et al*, 1995; Pitti *et al*, 1996; Pan *et al*, 1997; Peter *et al*, 1998). Following ligand binding, the receptors oligomerize and the pro-apoptotic molecules TRADD, FADD and FLICE/caspase-8 are recruited to their intracellular death domain forming the ‘death-inducing signalling complex’ (DISC) (Krammer, 1999). The subsequent events leading to apoptosis depend on the specific cell type being challenged. In type I cells the bulk induction of caspase-8 at the DISC leads to the direct activation of the effector caspase 3. In type II cells only little amounts of caspase-8 are activated at the DISC requiring the pro-apoptotic mitochondrial amplification loop for efficient caspase-3 activation (Scaffidi *et al*, 1998).

Resistance to apoptosis allows cancer cells to escape both the host immune response and conventional radio- and chemotherapy thus promoting tumour growth and progression. Several mechanisms have been proposed to negatively interfere with death receptor signal transduction at either the receptor or mitochondrial level. For some tumour cells the absence of CD95 (Butler *et al*, 1998; Loro *et al*, 1999) or TRAIL-R1 and TRAIL-R2 (Ozoren *et al*, 2000; Nguyen *et al*, 2001) at the cell surface has been reported. Additionally, two other TRAIL-receptors were discovered which are unable to transduce the death signal and are believed to negatively influence TRAIL-mediated apoptosis. TRAIL-R3 lacks the entire cytoplasmic domain and was proposed to act as a decoy receptor to inhibit TRAIL signalling (Degli-Esposti *et al*, 1997b; Walczak *et al*, 1997). TRAIL-R4 lacks only the death domain and its truncated cytoplasmic domain induces NF-*κ*B (nuclear factor *κ*B), which can protect cells from apoptosis (Degli-Esposti *et al*, 1997a). Moreover, TRAIL-R1 and TRAIL-R2 both are able to activate NF-*κ*B (Schneider *et al*, 1997) and inhibition of NF-*κ*B augmented TRAIL-mediated apoptosis in haematopoietic (Jeremias *et al*, 1998) and pancreatic carcinoma cells (Trauzold *et al*, 2001). The activation of NF-*κ*B has been shown to induce the expression of apoptosis-protecting proteins like IAP (inhibitors of apoptosis), TRAF2 and FLIP (Wang *et al*, 1998; Micheau *et al*, 2001). Constitutive downregulation of caspase-8 or the over-expression of FLIP represent other protective mechanisms preventing an efficient DISC formation (Irmler *et al*, 1997; Irisarri *et al*, 2000; Eggert *et al*, 2001; Fulda *et al*, 2001). For type II cells it has been shown that both overexpression of Bcl-x_L_ (Hinz *et al*, 2000) and induction of PKC by PMA (Scaffidi *et al*, 1999) are able to inhibit the cytochrome C release thus preventing the activation of caspase-9 and caspase-3.

The majority of pancreatic adenocarcinoma cell lines is resistant to CD95 and TRAIL-mediated apoptosis despite expressing the corresponding death receptors at the cell surface (Ungefroren *et al*, 1998; Trauzold *et al*, 2001). Recently, we revealed an anti-apoptotic role of constitutively overexpressed FAP-1 (Ungefroren *et al*, 2001) and Bcl- x_L_ (Hinz *et al*, 2000) in these cells. Moreover, we reported the existence of another, inducible resistance-mechanism operating in these cells: upon ligand binding, both CD95 and TRAIL-R activated protein kinase C (PKC) which protected pancreatic tumour cells from apoptosis by preventing the loss of mitochondrial membrane potential and cytochrome C release as well as by activation of NF-*κ*B (Trauzold *et al*, 2001).

Since pancreatic adenocarcinoma is one of the most aggressive cancer types with an extremely poor prognosis, it is crucial to reveal common characteristics of pancreatic tumour cells in order to develop new therapeutic strategies. Thus, we determined the expression levels of several apoptosis-relevant proteins in a total of five pancreatic tumour cell lines showing different potential to undergo apoptosis, and we functionally analysed these data in the context of sensitivity to anti-CD95- or TRAIL-induced cell death.

## MATERIAL AND METHODS

### Cell lines and culture conditions

Human pancreatic adenocarcinoma cell lines Capan1, Colo357, PancTuI, Panc89, Panc1, and their suppliers have been described previously (Sipos *et al*, 2003). All cells were cultured in RPMI 1640 supplemented with 10% FCS, 2 mM glutamine and 1 mM sodium pyruvate. Retrovirally transduced cells expressing Bcl-x_L_ and Bid as well as the corresponding vector controls received puromycin (2.5 *μ*g ml^−1^, Sigma, Deisenhofen, Germany) in addition. Colo357 cells over-expressing dominant negative FADD or transduced with an empty vector were cultivated in the presence of G418 (600 *μ*g ml^−1^). Capan1/XIAP and Capan1/pcDNA3-transfectants were grown in medium supplemented with 400 *μ*g ml^−1^ of G418.

For induction of apoptosis, agonistic monoclonal anti-CD95-antibodies clone CH11 (100 ng ml^−1^; Coulter Immunotech, Hamburg, Germany) or recombinant TRAIL (100 ng ml^−1^; R&D Systems GmbH, Wiesbaden, Germany) was added to the culture medium for 24 h.

### Expression vectors

Construction of the retroviral Bcl-x_L_ expression vector was described in detail earlier (Hinz *et al*, 2000). A cDNA encoding human Bid was generated by RT–PCR using total RNA from Colo357 cells, *Pfu* polymerase (Stratagene, Heidelberg, Germany) and primers Bid-forward (5′-ACCATGGACTGTGAGGTCAACAACG-3′, start codon underlined) and Bid-reverse (5′-TGGAACTGTCCGTTCAGTCCATCC-3′, stop codon underlined). The resulting fragment was inserted in sense orientation into the *Sna*BI site of the retroviral vector pBABE-puro. The correct orientation and the identity with the published nucleotide sequence were verified by sequencing. The cDNA for dominant negative (DN) FADD (clone AU1-NFD4, kindly provided by Dr. C. Vincenz, University of Michigan, Ann Arbor, USA) was released from pcDNA3 (Invitrogen, Karlsruhe, Germany) with *Hind*III and *Xho*I followed by polishment of both ends with Klenow fragment and subcloning into the *Pme*I site of the retroviral vector TJBA5bMolink-neo (Howard *et al*, 2000). These constructs were co-transfected into 293T producer cells along with retroviral packaging vectors as described previously (Howard *et al*, 2000). Retroviral particles were used for transduction of Colo357 (Bcl-x_L_, DN-FADD) or PancTuI and Panc89 (Bid) cells as described before (Hinz *et al*, 2000).

The XIAP c-DNA in pcDNA3 was a kind gift from Dr. K. Pfizenmaier (Inst. F. Zellbiologic and Immunologic, Stuttgart, Germany) and was transfected in the presence of LipofectAMIN 2000 (20 *μ*g ml^−1^, Invitrogen Corp., Eggenstein, Germany) into Capan1 cells.

### JAM-Assay

The JAM assay was performed as previously described (Ungefroren *et al*, 1998). The percentage of the target cell viability measured as percentage of high molecular weight DNA retained on glass fiber filters was calculated as: % viability=(E/S) × 100, where E (experimental) is cpm of retained DNA in the presence of apoptosis-inducing agent under study and S (spontaneous) is cpm of retained DNA of untreated control cells.

### FACS (fluorescence activated cell sorting) analysis

For detection of cell-surface expression of death receptors, 4 × 10^5^ EDTA-released cells obtained from monolayer cultures were incubated with anti-TRAIL-R1-4 (kindly provided by Dr. Henning Walczak, German Center for Cancer Research, Heidelberg, Germany) or anti-CD95 (APO1-3, Alexis Biochemicals, Grünberg, Germany) mouse mabs (1 : 100 in PBS/1% BSA) for 1 h at 4°C, washed twice in ice-cold PBS containing 0.05% sodium azide (PBS/azide) and incubated with anti-mouse biotin-conjugated (for TRAIL-receptors) and with FITC-conjugated anti-mouse secondary antibodies (for CD95) for additional 1 h at 4°C in the dark. After two further washes with PBS/azide, cells were re-suspended in 300 *μ*l 1% paraformaldehyde in PBS prior to FACS analysis (for CD95) or incubated with phycoerythrin-conjugated streptavidin (for TRAIL-receptors), washed again twice as before and re-suspended in 300 *μ*l 1% paraformaldehyde in PBS.

Flow cytometric analysis was carried out using a FACScan (Becton Dickinson, Heidelberg, Germany) and analysed with the Cell Quest program. At least 10 000 cells were examined for each determination with the window set to exclude cellular debris and non-viable cells.

As control the fluorescence of cells incubated with appropriate isotype matched antibodies followed by labelling with the corresponding secondary antibodies was measured.

### Western blot

For detection of the expression of apoptosis relevant proteins, cells were grown to 70% confluence and lysed in RIPA buffer. The lysates (30 *μ*g total protein per lane) were separated by SDS–PAGE, blotted onto PVDF-membrane and incubated with the appropriate primary antibodies followed by incubation with the HRP-conjugated secondary antibody (Amersham Pharmacia Biotech, Freiburg, Germany). Antigen visualization was performed by enhanced chemiluminescence (ECL-kit, Amersham Pharmacia Biotech). Primary antibodies used and their suppliers were as follows: anti-caspase-8 and anti-caspase-9 (StressGen Biotechnology, Canada), anti-caspase-3, anti-caspase-2, anti-caspase-7, anti-FADD, anti-Bcl-x_L_, anti-Apaf-1, anti-BAD (Transduction Laboratories/BD, Heidelberg, Germany), anti-Bfl-1 (Santa Cruz Biotechnology, Santa Cruz, CA), anti-Bid, anti-c-FLIP, anti-XIAP, anti-c-IAP2 and anti-Survivin (R&D Systems, Wiesbaden, Germany), anti-Smac/DIABLO (Biocarta, Hamburg, Germany) and anti-Bax (PharMingen, Hamburg, Germany).

## RESULTS

CD95- and TRAIL-mediated apoptosis and the cell surface expression of death receptors on pancreatic adenocarcinoma cell lines.

In this report we determined in parallel the sensitivities of five pancreatic carcinoma cell lines, Capan1, Colo357, PancTuI, Panc89 and Panc1 to anti-CD95- and TRAIL-mediated apoptosis in the context of the cell surface expression of the corresponding receptors. Here we used a concentration of 100 ng ml^−1^ of either CH11 or TRAIL because the recently published dose response curves for both stimuli and cell lines Colo357, PancTuI and Panc1 showed no significant further apoptosis at concentrations up to 1000 ng ml^−1^ (Hinz *et al*, 2000). Table 1

**Table 1:** Sensitivity of pancreatic tumour cells to CD95-mediated apoptosis.

**Cell line**	**CH11 viability (%)**	**TRAIL viability (%)**
Capan1	32.8±0.8	13.7±1.4
Colo357	66.6±1.5	31.9±0.7
PancTuI	95.7±4.1	86.7±4.0
Panc89	86.7±2.4	64.4±2.7
Panc1	98.6±0.3	75.4±3.0

Cells were treated for 24 h with agonistic anti-CD95 antibody CH11 or with recombinant TRAIL. The viability of the cells was determined by JAM-Assay. Data represent means ± SD of three independent experiments performed 6-fold each

shows that three cell lines were either almost completely (PancTuI and Panc1) or highly (Panc89) resistant to CD95-mediated apoptosis. Two other cell lines Colo357 and Capan1 were moderate and highly sensitive to anti-CD95-treatment, respectively.

Obviously, TRAIL provided stronger apoptotic stimuli than anti-CD95 since only the cell line PancTuI remained highly refractory (13.3% apoptosis) to TRAIL treatment whereas 24.6% of Panc1 cells, 35.6% of Panc89 cells, 68.1% of Colo357 cells and 86.3% of Capan1 cells died after 24 h exposition to TRAIL.

Despite the differences in sensitivity of Capan1 and Colo357 cells to anti-CD95 and TRAIL we classified these cell lines as rather sensitive in contrast to PancTuI, Panc89 and Panc1 cells, which were regarded as mostly resistant.

**Figure 1:**
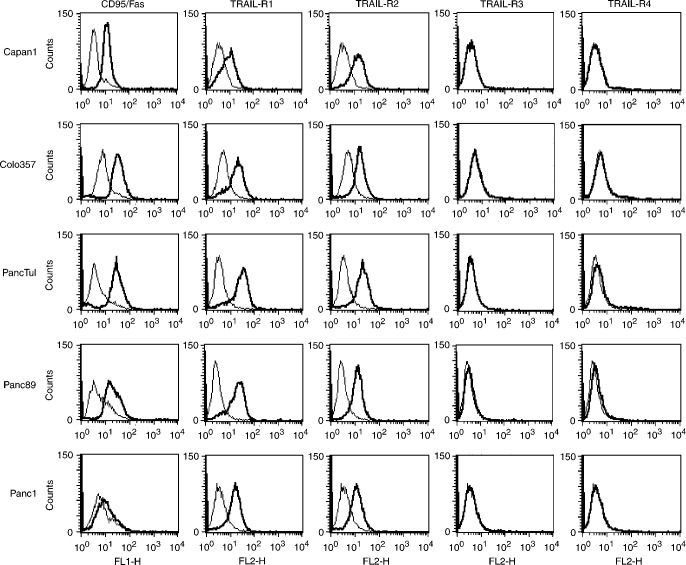
Flow cytometric analysis of cell surface expression of CD95, TRAIL-R1-4 on pancreatic tumour cells. Bold lines: specific monoclonal antibodies as indicated in the figure, thin lines: isotype control monoclonal antibodies. Shown are representative results of one out of three experiments performed.

Since the susceptibility of cells to anti-CD95 and TRAIL could reflect the different amount of corresponding receptors, we analysed the expression of CD95 and TRAIL-R1-4 at the cell surface of all five cell lines. Figure 1

shows that Capan1, Colo357 and PancTuI cells expressed comparable amounts of CD95 whereas the amount of CD95 on plasma membrane of Panc89 cells was slightly lower and on Panc1 cells almost undetectable. In contrary, TRAIL-R1 and TRAIL-R2 were highly expressed on all cells and even stronger on the most resistant PancTuI than on most sensitive Capan1 cells.

Recently, it has been shown that the sensitivity to TRAIL may be modulated by the expression of TRAIL-R3 and TRAIL-R4. For both receptors a protective role in TRAIL-mediated apoptosis has been postulated. Appropriate FACS analysis revealed no detectable levels of TRAIL-R3 on either cell line and no (Capan1, Colo357 and Panc1) or almost no detectable levels of TRAIL-R4 on PancTuI and Panc89 cells (Figure 1).

These data strongly suggest that the block in CD95- (except for Panc1 cells) and TRAIL-mediated apoptosis is located downstream of the receptor level.

### Expression of apoptosis-relevant proteins in pancreatic adenocarinoma cells

Since sensitivity to death receptor-induced apoptosis in pancreatic tumour cell lines did obviously not correlate with cell surface expression of death receptors, we systematically analysed the expression of a set of intracellular proteins known to be involved in transmission or inhibition of the death signal.

**Figure 2:**
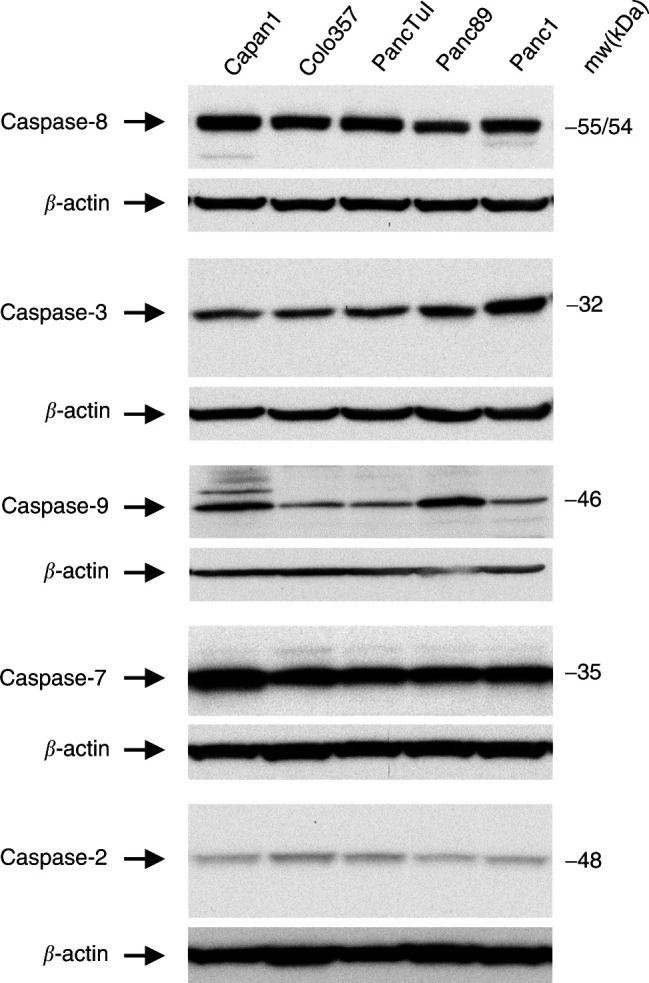
Expression of caspases in pancreatic adenocarcinoma cell lines. 30 *μ*g of whole cell lysates from Capan1-, Colo357-, PancTuI-, Panc89- and Panc1 cells were separated in 12% SDS–PAGE and analysed by Western blot using anti-caspase-8, anti-caspase-3, anti-caspase-9, anti-caspase-2, and anti-caspase-7 antibodies. Equal loading of the gels was verified by the determination of *β*-actin expression. Data are representative of at least three independent experiments.

Recently, it has been reported that deficiency in caspase-8 expression was a key determinant of apoptosis sensitivity of malignant brain tumours and melanoma (Fulda *et al*, 2001). In contrast, sensitive as well as resistant pancreatic tumour cells all expressed comparable and rather high amounts of caspase-8 (Figure 2

). Similarly, expression of caspase-9, -3, -7, and -2 was readily detectable in all cells although the levels of caspase-8, -9, and -7 were elevated in the most sensitive Capan1 cells (Figure 2).

**Figure 3:**
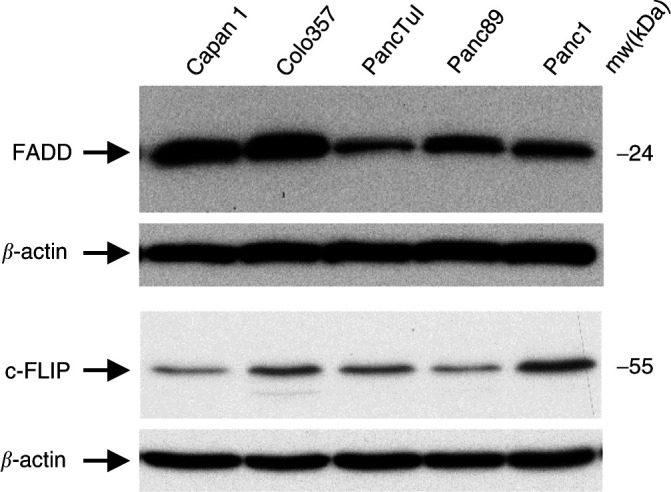
Expression of DISC interacting proteins FADD and FLIP in pancreatic tumour cells. Equal amounts of whole cell lysates (30 *μ*g) from each cell line were separated in 4–12% gradient gels and blotted onto PVDF membrane. Subsequently, the expression of FADD and FLIP was detected using specific antibodies. Equal loading of slots was verified by determination of *β*-actin expression. Data are representative of at least three independent experiments.

We found that the DISC interacting protein FADD was clearly down-regulated in resistant cells (Figure 3

). In addition, compared to Capan1, these cells expressed elevated levels of c-FLIP.

**Figure 4:**
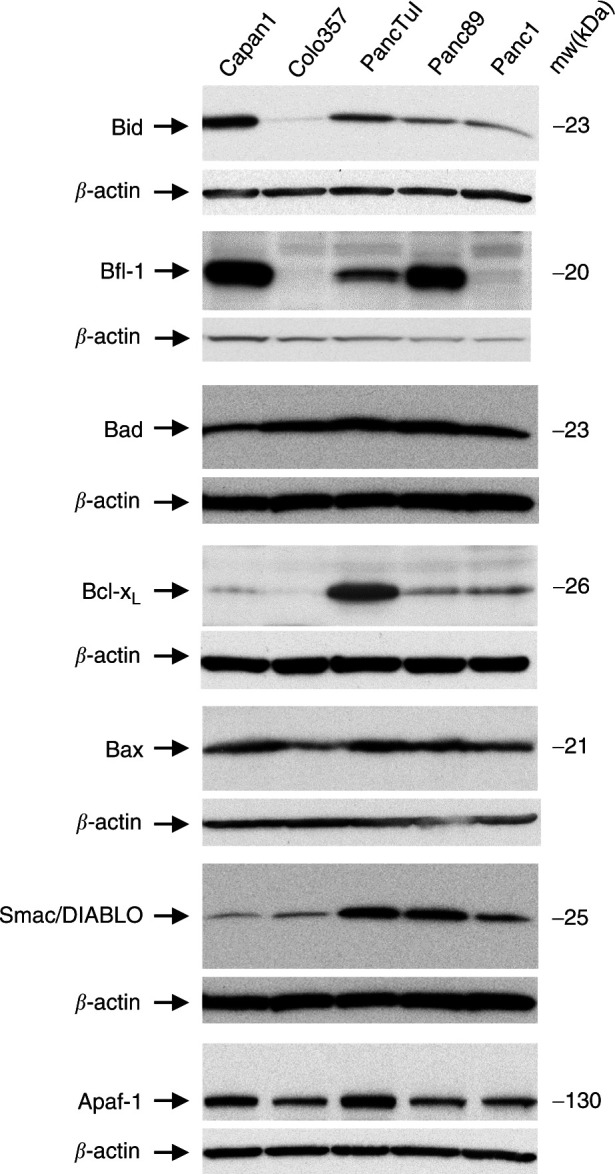
Expression levels of proteins involved in the mitochondrial apoptosis pathway in pancreatic adenocarcinoma cells. 30 *μ*g of whole cell lysates were analysed by Western blot analysis for constitutive expression of Bid, Bfl-1, Bad, Bcl-x_L_, Bax, Smac/DIABLO and Apaf-1. Equal gel loading was verified by assessing *β*-actin levels. Data are representative of three independent experiments.

Since it was shown (Hinz *et al*, 2000; Trauzold *et al*, 2001) that pancreatic tumour cell lines behave like type II cells in both, CD95- and TRAIL-mediated apoptosis, we did not only analyse the expression levels of caspases and DISC components, but also of proteins which participate in the mitochondrial apoptosis pathway (Figure 4

). The expression pattern of two of them, Bid and Bcl-x_L_ correlated well with the sensitivity of cell lines to TRAIL and anti-CD95-treatment. The pro-apoptotic Bid protein was clearly down-regulated in resistant cells in comparison to Capan1. But interestingly, moderately sensitive Colo357 cells also showed a strong down-regulation of Bid expression. In contrast, the anti-apoptotic Bcl-x_L_ was up-regulated in all resistant cells. Only recently Werner *et al*. demonstrated that the novel anti-apoptotic member of the Bcl-2 family Bfl-1 sequesters truncated Bid thereby inhibiting the mitochondrial pathway of apoptosis (Werner *et al*, 2002). Therefore, we additionally analysed the expression of Bfl-1. We found Bfl-1 to be clearly overexpressed in Capan1, Panc89 and PancTuI whereas Panc1 cells showed only very little and Colo357 cells no expression of Bfl-1 (Figure 4).

During apoptosis, the Smac/DIABLO protein is released from mitochondria along with cytochrome C, binds to and inhibits IAPs and promotes cytochrome C-dependent caspase-9 activation (Du *et al*, 2000). Analysis of Smac/DIABLO expression in pancreatic tumour cells surprisingly showed a reverse correlation with the sensitivity of these cells to anti-CD95 or TRAIL (Figure 4).

Since particular differences in the expression of caspases between sensitive and resistant cell lines were not observed, we also analysed the expression of several members of the IAP (inhibitors of apoptosis) family (reviewed by Deveraux and Reed, 1999). These proteins are direct inhibitors of caspases and thus play an apoptosis-protective role if up-regulated.

**Figure 5:**
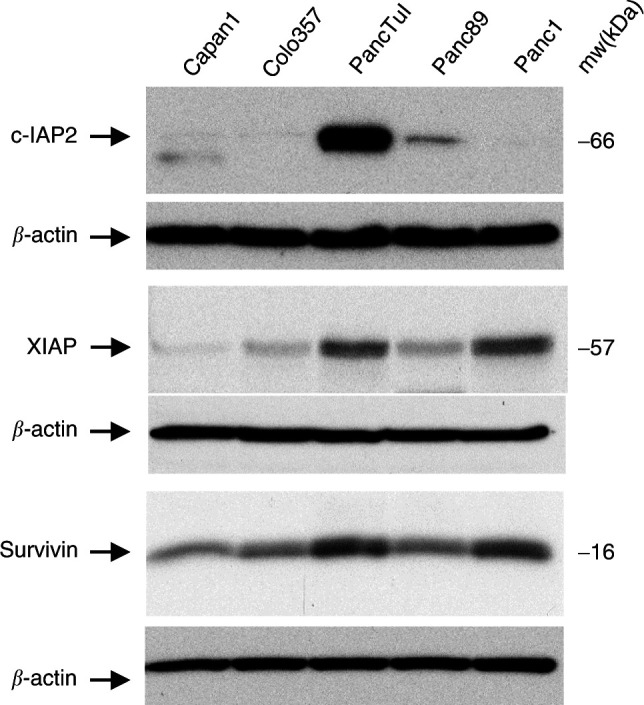
Immunoblot detection of proteins of the IAP-family in pancreatic tumour cells. Protein extracts (30 *μ*g) of pancreatic tumour cells were separated by 12% SDS–PAGE, blotted onto PVDF membranes and the expression of c-IAP2, XIAP and Survivin was analysed using specific antibodies. In parallel, *β*-actin expression was determined in all lanes. Representative results of three independent experiments are shown.

Here we show that the most resistant PancTuI cells expressed strongly elevated levels of c-IAP2, XIAP and Survivin (Figure 5

). Similarly, Panc1 cells which were also resistant to both, anti-CD95 and TRAIL, expressed more of IAP-proteins (XIAP, Survivin) than the sensitive Capan1 and Colo357 cells. Panc89 cells, which were resistant against anti-CD95 but only moderately resistant to TRAIL, expressed clearly less of all IAPs than PancTuI and Panc1 cells but still more than Capan1 or Colo357.

### Functional verification of the expression data

**Figure 6:**
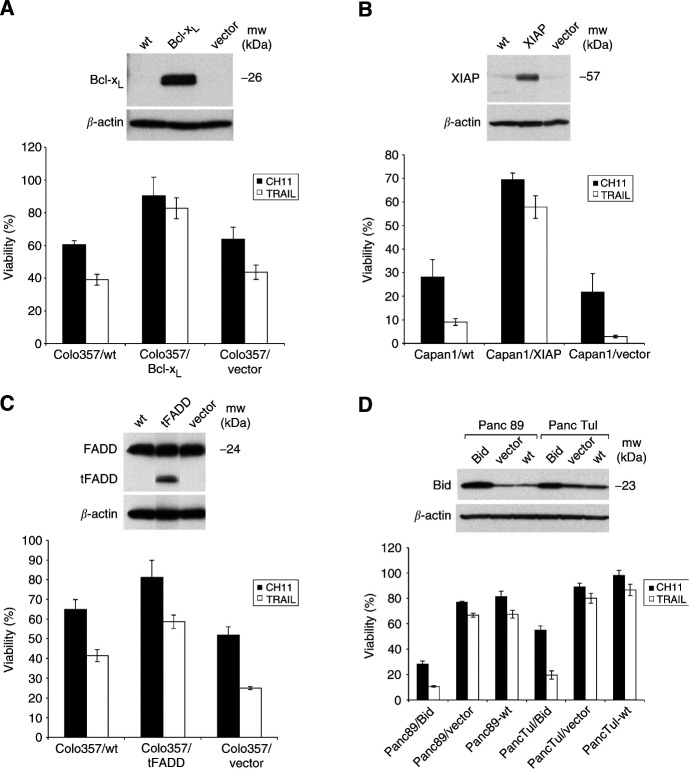
Anti-CD95 and TRAIL sensitivity of pancreatic adenocarcinoma cells overexpressing Bcl-x_L_ (**A**), XIAP (**B**), truncated FADD (tFADD) (**C**) and Bid (**D**). Upper parts of each figure show the expression of Bcl-x_L_, XIAP, tFADD or Bid determined by Western blot in cell pools retrovirally transduced (Bcl-x_L_, tFADD and Bid) or transfected (XIAP) with vectors bearing the corresponding genes (see material and methods). As control, wild-type-cells and cells transfected with an empty vector were analysed in parallel. Lower parts of each figure show the quantification of cellular DNA-fragmentation by the JAM-Assay in cell pools treated for 24 h with anti-CD95 (100 ng ml^−1^) or TRAIL (100 ng ml^−1^). Data represent means±SD (*n*=6) from one representative experiment out of at least three performed.

The Western blot analysis revealed over-expression of Bcl-x_L_ and XIAP and down-regulation of Bid as well as FADD in resistant cells. To verify the putative role of these proteins in the apoptosis deficiency of the respective pancreatic tumour cells, we constructed several recombinant cell lines either over-expressing those gene products found down-regulated, or over-expressing dominant-negative gene products found to be up-regulated in pancreatic tumour cells. Recombinant Colo357-Bcl-x_L_ transductants strongly over-expressed Bcl-x_L_ protein (Figure 6A

upper panel). These cells showed highly enhanced viability after anti-CD95 or TRAIL treatment compared to apoptosis-sensitive Colo357 wildtype (WT) cells.

Similarly, an over-expression of XIAP in the most sensitive pancreatic tumour cell line Capan1 dramatically increased their viability after treatment with both death-inducing ligands (Figure 6B).

Since Colo357 as well as Capan1 cells expressed high amounts of FADD, and all resistant cells showed strongly reduced levels of FADD, we investigated if we could lower the sensitivity of Colo357 cells to anti-CD95 and/or TRAIL by the expression of a dominant negative FADD protein. This mutant protein carries a deletion of the N-terminal portion of the protein known as the death effector domain (DED) and thus attenuates its ability to induce cell death (Chinnaiyan *et al*, 1995). As shown in Figure 6C Colo357 cells transduced with an expression vector coding for the dominant negative FADD mutant (expressing the truncated tFADD) were markedly more resistant to anti-CD95 and TRAIL than wildtype Colo357 or cells transduced with an empty vector.

Similarly, the down-regulation of Bid, also plays a role in establishing the resistant phenotype, since the reconstitution of the high Bid expression in PancTuI and Panc89 cells led to sensitisation of both cell lines to CD95- and TRAIL-mediated apoptosis (Figure 6D).

## DISCUSSION

In this study we identified several changes in the expression pattern of apoptosis-relevant proteins in mostly resistant versus rather sensitive pancreatic tumour cells. We observed a concerted down-regulation of several pro-apoptotic proteins in conjunction with the up-regulation of anti-apoptotic proteins in resistant cells. We showed that all pancreatic tumour cells expressed TRAIL-R1 and TRAIL-R2 irrespective of their sensitivity to recombinant TRAIL. Moreover, we could not detect TRAIL-R3 on the surface of Capan1, Colo357, PancTuI and Panc1 cells and we found only extremely low level of this receptor on Panc89 cells. Similarly, TRAIL-R4 was undetectable on Capan1, Colo357 and Panc89 cells and almost undetectable on PancTuI and Panc89 cells. Thus in the case of the TRAIL-mediated apoptosis the resistance seemed to be achieved downstream of the receptor level. In the case of CD95 we found some heterogeneity in the CD95-expression on the cell surface of apoptosis-resistant cells. Panc1 cells expressed extreamly little CD95 whereas the expression of CD95 on PancTuI cells was similar and on Panc89 cells slightly lower compared to the sensitive Capan1 or Colo357 cells. The observed down-regulation of CD95 may indeed play an important role in biological aggressiveness of pancreatic tumours, since just recently Bernstorff *et al*. (2002) reported a reduced membranous CD95 staining in tissue sections of invasive ductal adenocarcinomas compared to normal pancreatic tissue.

The results presented here suggest that tumour cells acquired resistance against anti-CD95 and TRAIL by modifying the expression of several apoptosis-relevant proteins beginning with those acting already at the receptor level. Here, additionally to the perturbations of the receptor expression, proteins interfering with the DISC formation are involved. Thus we found strongly diminished amounts of FADD in resistant cells compared to the sensitive ones. Since FADD is an adaptor protein responsible for the recruitment of pro-caspase-8 to the death receptor, its down-regulation will affect the DISC formation and in consequence the apoptotic behaviour of the cells. This hypothesis was proven by transfection of Colo357 cells with a dominant negative FADD construct (Figure 6C). As an additional mechanism of apoptosis inhibition serves the increased synthesis of FLIP observed mostly pronounced in Panc1, and to a lesser extend in Colo357 and PancTuI cells. This in conjunction with reduced FADD expression clearly contributes to the acquired apoptosis resistance of pancreatic tumour cells. The importance of the balance between anti-apoptotic and pro-apoptotic molecules for the apoptotic outcome is supported by the observation that Colo357 cells being less sensitive than Capan1 express slightly higher amount of FADD, which may than be counteracted by an increased expression of FLIP.

In contrast to brain tumour cells the expression of caspases is not limiting for apoptosis of pancreatic tumour cells and does not correlate with the sensitivity status of the cells under study expect for most sensitive Capan 1 cells, which expressed elevated levels of caspase-8, -9, and -7. Interestingly, Panc1- or Panc89 cells, both classified as mostly resistant, expressed slightly elevated levels of caspase-3 or caspase-9, respectively.

Pancreatic carcinoma cells are regarded as type II cells in which only small amounts of active caspase-8 are generated at the DISC and a mitochondrial amplification loop is necessary for proper apoptosis induction (Hinz *et al*, 2000; Trauzold *et al*, 2001; Ungefroren *et al*, 2001).

Following caspase-8-activation the full length Bid, a small pro-apoptotic cytosolic protein, is cleaved and the p15 tBid fragment targets mitochondria eventually leading to the disruption of the mitochondrial transmembrane potential Δψm and cytochrome C release (reviewed by Yin, 2000). Cytochrome C associates with Apaf-1 and induces its oligomerization. Oligomerized Apaf-1 binds the pro-caspase-9 forming the so-called apoptosome, which triggers the autocatalytic processing of procaspase-9. Active caspase-9 cleaves and activates the effector caspases-3, -6 and -7, which in turn, by cleaving their substrates, complete the apoptotic cell death. Along with cytochrome C the Smac/DIABLO protein is released into the cytoplasm and binds to IAPs, thereby inhibiting them, and consequently allowing the caspases to complete the apoptotic process (Srinivasula *et al*, 2000). Bcl-x_L_ is an anti-apoptotic protein, which similarly to Bcl-2 prevents the mitochondrial membrane disruption (reviewed by Korsmeyer, 1999). It has been shown that Bcl-x_L_ can protect cells from apoptosis induced by death receptors and chemotherapeutic agents. We show here that Bid is down-regulated and Bcl-x_L_ is up-regulated in resistant pancreatic tumour cell lines. Such perturbation in the expression of both proteins are likely to contribute to the resistant phenotype since these cells require the mitochondrial amplification loop for efficient induction of anti-CD95 and TRAIL-induced apoptosis. In fact, we could recently demonstrate a protective role of Bcl-x_L_ in CD95- and TRAIL-R-mediated apoptosis (Hinz *et al*, 2000). Data of the present work concerning the role of Bid down-regulation support this hypothesis, since stable over-expression of Bid in resistant PancTuI and Panc89 cells converted both into highly sensitive cell lines. The observed strong down regulation of Bid in sensitive Colo357 cells is accompanied with nearly absence of the expression of Bcl-x_L_ as well as Bfl-1. Bfl-1 is a protein of the Bcl-2 family, which has recently been shown to bind and inactivate truncated Bid (Werner *et al*, 2002). Thus no or very low expression of Bfl-1 in Colo357 cells in conjunction with very little amounts of Bcl-x_L_ may account for the observed sensitivity of these cells to CD95- and TRAIL-mediated apoptosis. Unexpectedly, the most sensitive Capan1 cells expressed the highest level of Bfl-1. This must be considered in the context of the very high amount of Bid and strongly diminished expression of Bcl-x_L_. In addition, the high expression of caspases and FADD in combination with a low expression of FLIP may contribute to the high sensitivity of Capan1 cells towards CD95- and TRAIL-mediated apoptosis.

An additional level known to influence the apoptosis sensitivity is represented by the regulation of caspase activation. Members of IAPs family e.g. c-IAP1, c-IAP2, XIAP and Survivin all bind to and potently inhibit caspases-3, -7 and -9. XIAP has been shown to be ubiquitously expressed in adult and fetal tissues (Liston *et al*, 1996), whereas c-IAP1 and c-IAP2 expression is more heterogeneous being abundant in kidney, liver, lung and small intestine and low in tissues of the central nervous system, pancreas, peripheral leukocytes and the mammary gland (Young *et al*, 1999). The constitutive expression of Survivin has been reported to be restricted to fetal tissues, but interestingly, a high level of expression was observed in several human cancers (Ambrosini *et al*, 1997). The analysis of its promoter region and *in vitro* data suggested that Survivin is a G2/M associated protein involved in the control of proliferation. Analysis of the expression of IAPs in pancreatic tumour cells revealed the increased levels of XIAP and Survivin in all three resistant cell lines and, in particular, a very strong overexpression of c-IAP2 in the most resistant PancTuI cells. Both mostly sensitive cell lines showed reduced expression of IAPs with the lowest levels of all IAPs in Capan1 cells.

The results presented here reveal multiple changes of the expression of apoptosis-relevant proteins in resistant pancreatic adenocarcinoma cell lines. Concurring observed decreased levels of FADD, elevated levels of FLIP, and in one case down-regulation of the CD95 receptor all suggest that protection from death receptor mediated apoptosis occurs at least partially already at the receptor level by inhibition of DISC formation. Additionally, a decrease of Bid and an increase of Bcl-x_L_ expression impede the mitochondrial amplification loop essential for apoptosis in type II cells. The expression of caspase inhibitors XIAP, c-IAP2 and Survivin provides additional protection. The most resistant PancTuI cells accumulated more changes in the expression of apoptosis relevant proteins than less resistant Panc89 or Panc1. Correspondingly, the expression pattern of these proteins in Colo357 and Capan1 cells reflects the differences in the apoptosis susceptibility. In conclusion, concerted multiple changes rather than single or few perturbations of death receptor signalling are likely to be responsible for the resistance of pancreatic adenocarcinoma cells to CD95- and TRAIL-R-mediated apoptosis.
